# Psychometric evaluation of the WHODAS 2.0 and prevalence of disability in a Swedish general population

**DOI:** 10.1186/s41687-023-00580-0

**Published:** 2023-04-05

**Authors:** Paulina Norén, Jan Karlsson, Emma Ohlsson-Nevo, Margareta Möller, Liselotte Hermansson

**Affiliations:** University Health Care Research Center, Faculty of Medicine and Health, Örebro University, Örebro, Sweden

**Keywords:** Disability evaluation, Health surveys, Patient-reported outcome measures, RAND-36, Reference values, Validity and reliability

## Abstract

**Background:**

The World Health Organization Disability Assessment Schedule (WHODAS 2.0) is a generic questionnaire that captures health and disability-related functioning information corresponding to six major life domains: *Cognition*, *Mobility*, *Self-care*, *Getting along*, *Life activities*, and *Participation*. The WHODAS 2.0 is used in a wide range of international clinical and research settings. A psychometric evaluation of WHODAS 2.0, Swedish version, in the general population is lacking, together with national reference data to enable interpretation and comparison. This study aims to evaluate the psychometric properties of the Swedish 36-item version of WHODAS 2.0 and describe the prevalence of disability in a Swedish general population.

**Methods:**

A cross-sectional survey was performed. Internal consistency reliability was assessed with Cronbach’s alpha. The construct validity was evaluated with item-total correlation, Pearson’s correlation between the WHODAS 2.0 domains and the RAND-36 subscales, analysis of known groups by one-way ANOVA, and analysis of the factor structure by confirmatory factor analysis.

**Results:**

Three thousand four hundred and eighty two adults aged 19–103 years (response rate 43%) participated. Significantly higher degrees of disability were reported by the oldest age group (≥ 80 years), adults with a low level of education, and those on sick leave. Cronbach’s alpha was from 0.84 to 0.95 for the domain scores and 0.97 for the total score. The item-scale convergent validity was satisfactory, and the item-scale discriminant validity was acceptable except for the item about sexual activity. The data partially supported the factor structure, with borderline fit indices.

**Conclusion:**

The psychometric properties of the self-administered Swedish 36-item version of the WHODAS 2.0 are comparable to those of other language versions of the instrument. Data of the prevalence of disability in Swedish general population enables normative comparisons of WHODAS 2.0 scores of individuals and groups within clinical practice. The instrument has certain limitations that could be improved on in a future revision. The test–retest reliability and responsiveness of the Swedish version of WHODAS 2.0 for different somatic patient populations remain to be evaluated.

**Supplementary Information:**

The online version contains supplementary material available at 10.1186/s41687-023-00580-0.

## Background

There is a need for international comparable and relevant data on functioning and disability [1]. Self-reported information on disability is thought to contribute an important perspective on the functioning of individuals. People sometimes experience disability in a way that is contrary to the expectations of, for example, health care professionals; they may also report a good or excellent quality of life despite limitations in their functioning [2]. Through the use of patient-reported outcome measures, systematic knowledge regarding patients’ own perspectives on health or health-related concerns can be obtained. The systematic use of these measures in clinical practice has evolved over decades, and a vast number of different self-report questionnaires have been designed [3, 4].

With the World Health Organization Disability Assessment Schedule (WHODAS) 2.0 [5], information on self-perceived disability categorized into six functioning domains, including that of social participation activities, can be obtained.

WHODAS 2.0 not only addresses the traditional aspects of a person’s primary activities but also includes a wider perspective that encompasses cognition, mobility, self-care, getting along, life activities (household and work/study) and participation. Participation or social participation has been highlighted by health policy as a core concept and an ultimate goal for functioning since the International Classification of Functioning, Disability and Health model was published in 2001 [6]. WHODAS 2.0 is based on this classification [7] and its Activity and Participation component and is considered the leading disability measure worldwide [8]. It is a generic instrument that can be used to assess disability in adults who have any disease or injury, regardless whether that disability is based on somatic, mental or substance-use disorders [5]. Therefore, the instrument is useful in many different settings [8, 9] and as part of the initial assessment of rehabilitation needs [10]. WHODAS 2.0 has been cross-culturally developed and translated into more than 47 languages and dialects [8, 11–14], which enables international use, comparisons between patient populations and meta-analyses [5].

In 2013 the Swedish National Board of Health and Welfare initiated the task of producing a suitable translation of the questionnaires and the manual, aiming to produce an official and widely accepted Swedish version of the WHODAS 2.0. The translation followed the WHODAS 2.0 Translation Package version 1.0 with forward translation, back translation, and linguistic evaluation in an iterative process. For the forward translation, a group consisting of an expert panel including health professionals (two occupational therapists and one psychiatrist), an expert on ICF terminology, and the head of a unit at the Swedish National Board of Health and Welfare participated. An independent translator blinded to the original English version performed the back translation which confirmed the equivalence between the Swedish translation and the original version. The final version of the Swedish WHODAS 2.0 is approved by the WHO and is, since 2015, available at the Swedish National Board of Health and Welfare [15].

Although WHODAS 2.0 has undergone several psychometric evaluations, it is necessary to perform further evaluations when a new language version has been developed [16]. The Swedish 36-item version of WHODAS 2.0 has been psychometrically evaluated in mixed psychiatric populations [17–19]. However, no studies have been conducted to determine the extent to which the Swedish 36-item version is valid for the wide range of its intended users in Sweden, and reference data from the Swedish general population are not available. Such data should be used as a reference when determining the extent of rehabilitation needs in patients. Furthermore, to improve clinical utility, reference data should describe the current natural occurrence of, e.g., disability, in the community [16, 20]. General population data for the 36-item version of WHODAS 2.0 have been reported for Taiwan [21, 22] and middle-aged and elderly individuals in Spain [23]; however, norm values applicable to the Swedish national setting do not exist. There is a lack of knowledge regarding the degree of disability in adults of different ages in the general Swedish population as measured by WHODAS 2.0. Therefore, the aim of this study was to evaluate the psychometric properties of the Swedish 36-item version of WHODAS 2.0 and describe the prevalence of disability in a Swedish general population.

## Methods

We used a population-based survey design. The data collection was part of a larger study presented earlier [24]. The study was granted ethical approval by the Regional Ethical Review Board of Uppsala (reference number 2015/071). All procedures were in accordance with the ethical standards of the regional research committee and with the Declaration of Helsinki 1964 and its later amendments. Informed consent was obtained from all individual participants included in the study.

### Participants and procedure

A random sample of 8140 adults from a general population in a central region of Sweden, stratified according to sex and age, were invited to participate. The sample size estimation was based on 80% power (α = 0.05) to detect a difference between groups by 10 scale points for WHODAS 2.0 and the RAND-36 Measure of Health-Related Quality of Life (RAND-36) [24].

A study-specific questionnaire containing demographic questions, the WHODAS 2.0 and the RAND-36, was used in the survey. Together with an informational letter and a prepaid envelope, the questionnaires were sent by regular mail in two separate mailings during 2015 and 2016. The invitations to participate were followed by a thank-you and reminder card two weeks later. A reminder letter and a prepaid envelope were sent to those who did not return the questionnaires after five weeks.

### Instruments

WHODAS 2.0 is a generic questionnaire that captures health and disability-related functioning information corresponding to six major life domains: *Cognition* (6 items), *Mobility* (5 items), *Self-care* (4 items), *Getting along* (5 items), *Life activities* (which is divided into two domains concerning household (4 items) and work/study (4 items), and *Participation* (8 items) [5]. All the questions relate to a respondent’s average experienced difficulties over the last 30 days and are answered on a five-point response scale with the following options: none, mild, moderate, severe, and extreme/cannot do. The WHODAS 2.0 is available as a 36-item (32 items are answered by those not working or studying) or a 12-item version and as a hybrid version of 12 + 24 items [5]. It can be administered through self-reporting, interviews, or proxy. For this study, the 36-item Swedish self-administered version was used [15]. The scores were calculated according to the complex scoring model [5] and converted to scores ranging from 0 (no difficulties/best possible functioning) to 100 (extreme difficulties/worst possible functioning); i.e., lower scores are positive. Missing items were addressed according to the manual [5]; i.e., two items were allowed to be unanswered when calculating the total score, but only one missing item was allowed when calculating each specific domain score. The missing item values were imputed using the mean score of the nonmissing items within the corresponding domain for each respondent. To compute a total score, domain scores for all the domains except the work/study portion of the *Life activities* domain were required. The responses to a 32-item version of WHODAS 2.0 for those not working or studying are considered comparable to the full 36-item version.

The RAND-36 includes the same items as the Medical Outcomes Study 36‐item Short‐Form health survey [25, 26] and is previously used for validation of new versions of WHODAS 2.0 [8]. In this study, the Swedish RAND-36 [27] was used to assess convergent and discriminant validity. The instrument comprises eight multi-item scales: Physical functioning (10 items), Role-functioning/physical (4 items), Pain (2 items), General health (5 items), Energy/fatigue (4 items), Social functioning (2 items), Role-functioning/emotional (3 items), and Emotional well-being (5 items). The scores were summed and converted into scales ranging from 0 (worst possible health) to 100 (best possible health). If at least half of the items in a scale were answered, a scale score was calculated. The missing items were imputed by using a person-specific mean value based on the non-missing items of each scale [26].

### Statistical analysis

The scale properties of WHODAS 2.0 were assessed by calculating floor/ceiling effects, missing data per item, domain and total scores. The items were considered feasible if the proportion of missing items was below 10% [28]. The floor/ceiling effects were considered if more than 15% of the respondents obtained the lowest or highest possible domain or total score [29]. Internal consistency reliability was calculated using Cronbach’s alpha. An alpha value of 0.7 or higher is considered acceptable for group comparison, while an alpha coefficient of ≥ 0.9 is recommended for individual assessment [16].

Construct validity was investigated by testing item convergent validity, i.e., the degree to which items within each domain were correlated (corrected for overlap). An item-scale correlation of at least 0.40 is considered adequate for item convergent validity [16]. For item discriminant validity, the items within a domain are expected to be more highly correlated with their own domain than with other domains. The convergent and discriminant validity of the WHODAS 2.0 domains were further examined through a correlation analysis of the RAND-36 subscales. To facilitate comparison with other WHODAS 2.0 validation studies, Pearson´s correlation coefficients were computed. Predefined hypotheses regarding the associations between all the WHODAS 2.0 domains and all the RAND-36 subscales were formulated by the first author based on the content of the items within each domain or subscale and discussed with the coauthors until consensus was achieved. A low correlation was defined as r < 0.3, a medium correlation as r = 0.3–0.6, and a high correlation as r > 0.6 [30].

The known-group validity was estimated by testing the significant differences between the age groups (20–29, 30–39, 40–49, 50–59, 60–69, 70–79, and 80+ years), levels of education, and main occupations with a one-way analysis of variance (ANOVA), followed by Tukey’s honestly significant post hoc test. The participants turning 20 years old within the year were categorized into the Age Group 20–29. The effect size (ES) of the significant differences between the subgroups was further analyzed with Cohen’s *d* [31], where positive ES represents increased disability. Due to the group size differences, the ES was determined by dividing the mean differences between the groups by an adjusted pooled standard deviation that was weighted for the sample size. A small difference was defined as *d* = 0.20–0.49, a moderate difference as *d* = 0.50–0.79, and a large difference as *d* ≥ 0.8 [32]. We hypothesized that the participants in the older age groups, those with a mandatory education and those on long-term sick leave or receiving old age pensions would report a higher degree of disability. The differences between females and males were analyzed with the Mann–Whitney U test.

The construct validity of the 32-item version of WHODAS 2.0 was finally analyzed through confirmatory factor analysis (CFA). Factor loadings ≥ 0.4 were considered adequate [33]. Acceptable model fit was defined as a comparative fit index (CFI) value close to or higher than 0.95, a Tucker Lewis index (TLI) of 0.95, a root mean square error of approximation (RMSEA) of 0.08 or lower, and a standardized root mean square residual (SRMR) of 0.08 or lower [30]. To enable comparisons with previous studies, this analysis was performed without the work/study items of the *Life activities* domain.

Disability percentiles were calculated for the total sample and for all age groups.

IBM SPSS statistics for Windows Version 22 and for CFA SAS 9.4 were used for statistical analysis.

## Results

The study had a response rate close to 43%, and 3482 adults aged 19–103 years from the general population in the region participated (Table 1). Nearly 55% of the participants were females.

**Table 1:** Demographic characteristics of the study participants

Total N (%)	3482 (100)
*Sex, n (%)*	
Female	1906 (54.7)
Male	1576 (45.3)
*Age*	
Mean (SD)	60 (20.2)
Median	57
Range	19–103 years
*Age group, n (%)*	
20–29	402 (11.5)
30–39	499 (14.3)
40–49	412 (11.8)
50–59	405 (11.6)
60–69	591 (17.0)
70–79	639 (18.4)
80 +	534 (15.3)
*Country of birth, n (%)*	
Sweden	3020 (88.7)
Other Nordic country	111 (3.2)
Other European Country	107 (3.1)
Outside of Europe	164 (4.7)
Missing	80 (2.3)
*Level of education, n (%)*	
Mandatory	810 (23.3)
High school	1114 (32.0)
University/Higher education	1103 (31.7)
Other	363 (10.4)
Missing	92 (2.6)
*Main occupation, n (%)*	
Employed/Own a company	1593 (45.7)
Student	154 (4.4)
Old age pension	1332 (38.3)
Activity or sickness compensation	100 (2.9)
Unemployed	80 (2.3)
Other	141 (4.0)
Missing	82 (2.4)

*SD* = standard deviation

The missing items were below the critical rate of 10%, except for the items within the *Life activities: work/study* domain (30.0–30.9%), as individuals not working or studying were instructed to skip these items. The missing items pertaining to the *Participation* domain and the *Getting along* domain item (D4.5) concerning sexual activities were close to the critical rate, ranging from 8.4 to 9.5% for *Participation* and 8.6% for item D4.5. This resulted in a missing domain score of 11% for the *Participation* domain (Table 2). The rate of missing responses for the other items ranged from 5.1 to 6.1%. Clear floor effects were noted; however, no ceiling effects were observed (Table 2).

**Table 2:** Distribution and internal consistency reliability of the WHODAS 2.0 domains and the total scores in a Swedish general population

WHODAS 2.0	n	Mean (SD)	Skewness	Kurtosis	Median	Observed range	Floor effect %	Ceiling effect %	Missing domain %	Cronbach’s α
Cognition	3221	14.0 (19.1)	1.565	1.973	5.0	0–100	42.9	0.2	7.5	0.92
Mobility	3248	15.0 (22.7)	1.622	1.790	0.0	0–100	51.8	0.4	6.7	0.93
Self-care	3255	7.6 (17.7)	2.894	8.426	0.0	0–100	74.5	0.2	6.5	0.88
Getting along	3213	16.2 (21.0)	1.396	1.447	8.3	0–100	46.3	0.3	7.7	0.84
Life activities: household	3263	18.9 (26.3)	1.360	0.957	0.0	0–100	52.3	1.7	6.3	0.96
Life activities: work/study	2364	15.4 (23.9)	1.771	2.624	0.0	0–100	55.1	2.0	32.1*	0.96
Participation	3089	18.4 (20.2)	1.193	0.786	12.5	0–100	30.0	0.0	11.3	0.91
Total score	2989	15.2 (17.5)	1.484	1.850	15.2	0–96.7	17.4	0.0	14.2	0.97

A Cronbach’s alpha value of 0.7 or higher is considered to be acceptable for group comparison, while a score ≥ 0.9 is required for individual comparison

A participant did not have a domain score if more than one item was missing in each domain: domain scores for all the domains except the work/study portion of the *Life activities* domain were needed to compute a total score

*Individuals who did not work or study were instructed to skip the items in this domain

The internal consistency reliability was acceptable for all the domains. The Cronbach’s alpha coefficients ranged from 0.84 to 0.96 for the domains, while a value of 0.97 was noted for the total score (Table 2). All but two of the domains, i.e., *Self-care* and *Getting along*, had alpha values acceptable for individual comparisons. The Cronbach’s alpha became weaker if any item was deleted, indicating that all the items within the domains are important to assess disability.

### Convergent and discriminant validity

The item-scale convergent validity was satisfactory for all the items (r ≥ 0.4). The item-scale discriminant validity was satisfactory for all the items except for the *Getting along* domain item D4.5 concerning sexual activities. This item had a similar correlation with the other domains (r = 0.36–0.52) as it had with *Getting along* (r = 0.43). Hence, it did not fulfill the criterion for item-scale discriminant validity.

The RAND-36 was used to test the convergent and discriminant validity on the domain level. The correlations between the WHODAS 2.0 domains and the RAND-36 subscales were generally as expected or higher than expected, and 37 of the 56 correlations (66%) were correlated consistently with our predefined hypotheses (Table 3).

**Table 3:** Pearson’s correlations between the WHODAS 2.0 domain scores and the RAND-36 scale scores

RAND-36	Cognition	Mobility	Self-care	WHODAS 2.0			
				Getting along	Life activities: Household	Life activities: Work/study	Participation
Physical functioning	− 0.40 L	− **0.77 H**	− **0.51 M**	− 0.42 L	− **0.57 M**	− **0.51 M**	− **0.58 M**
Role-functioning/physical	− 0.42 L	− 0.61 M	− **0.42 M**	− 0.41 L	− **0.56 M**	− **0.60 M**	− 0.65 M
Pain	− 0.39 L	− **0.58 M**	− **0.41 M**	− 0.37 L	− **0.50 M**	− **0.51 M**	− 0.61 M
General health	− **0.48 M**	− **0.56 M**	− **0.41 M**	− **0.48 M**	− **0.54 M**	− **0.54 M**	− 0.67 M
Energy/fatigue	− **0.49 M**	− **0.44 M**	− **0.35 M**	− **0.46 M**	− **0.54 M**	− **0.54 M**	− 0.63 M
Social functioning	− **0.56 M**	− 0.55 L	− 0.48 L	− **0.56 M**	− 0.63 M	− 0.65 M	− **0.76 H**
Role-functioning /emotional	− **0.49 M**	− 0.38 L	− **0.35 M**	− **0.47 M**	− **0.50 M**	− **0.53 M**	− **0.59 M**
Emotional well-being	− **0.54 M**	− 0.36 L	− 0.36 L	− **0.54 M**	− **0.49 M**	− **0.54 M**	− 0.63 M

The correlations marked in bold are consistent with the predefined hypotheses regarding low (L) r < − 0.3; medium (M) − 0.3 < r < − 0.6; or high (H) r > − 0.6 correlations. All the correlations were significant (*p* < 0.001)

RAND-36 = equal to the Medical Outcomes Study 36‐item Short‐Form health survey (SF-36). The predefined hypotheses are based on the expectation that some WHODAS 2.0 domains and RAND-36 subscales are expected to have low, medium or high correlations

### Known-groups validity

**Fig. 1:**
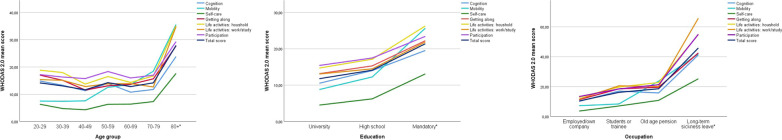
Normative data of the WHODAS 2.0 in different age, education and occupation. The mean domain and total scores for age groups (20–29, 30–39, 40–49, 50–59, 60–69, 70–79, 80+), level of education (reported as university, high school or mandatory studies) and main occupation (employed or own a company, students or trainees, old-age pension or long-term sick leave). A one-way analysis of variance (ANOVA), followed by Tukey’s honestly significant difference post hoc test was performed. *In the pairwise subgroup comparison the age group 80+ , mandatory level of education and main occupation reported as long-term sick leave were significant (*p* < 0.05, 95% CI)

In line with our expectations, the oldest age group (80+ years) had a significantly higher level of disability. A gradually increasing trend in disability among the participants in the older age groups only appeared for the *Mobility* domain. The disability levels of the adults younger than 80 years were more evenly distributed, ranging from 11.6 to 14.3 for the mean total scores and from 4.3 to 18.4 for the mean domain scores (Fig. 1, Additional file 1: Table S1). In contrast with all the other age groups (20–29, 30–39, 40–49, 50–59, 60–69, and 70–79 years), the ESs in the oldest age group (80+) ranged from moderate to large in terms of the total scores (*p* < 0.05, ES = 0.68–0.91) and small to large in terms of the domain scores (*p* < 0.05, ES = 0.44–1.27) (Fig. 1, Table 4). A higher degree of disability was reported for the subgroups with mandatory education and those on long-term sick leave (Fig. 1, Additional file 2: Table S2, Additional file 3: Table S3). The differences in total and domain scores between the subgroups receiving old-age pensions and students or trainees were not significant, except for the *Mobility* domain (*p* < 0.05, ES = 0.60) and the *Self-care* domain (*p* < 0.05, ES = 0.18) (Table 4). No significant differences were found between men and women.

**Table 4:** Effect size of the significant differences between the subgroups

	Subgroups for comparison*	WHODAS 2.0							
		Total score	Cognition	Mobility	Self-care	Getting along	Life activities: household	Life activities: work/study	Participation
		ES	ES	ES	ES	ES	ES	ES	ES
*Age group*									
20–29	80+	0.75	0.44	1.23	0.52	0.48	0.57	0.76	0.60
30–39		0.81	0.53	1.27	0.63	0.57	0.60	0.79	0.63
40–49		0.91	0.61	1.25	0.64	0.78	0.77	0.90	0.67
50–59		0.68	0.45	0.92	0.51	0.65	0.64	0.82	0.50
60–69		0.83	0.64	0.92	0.53	0.67	0.76	0.83	0.65
70–79		0.78	0.61	0.68	0.48	0.59	0.66	0.97	0.62
*Education*									
High school	Mandatory	0.40	0.27	0.57	0.36	0.32	0.34	0.29	0.28
University		0.56	0.47	0.77	0.48	0.43	0.44	0.37	0.40
*Occupation*									
Employment or own company	Student or trainee	0.43	0.44	–	–	0.39	0.34	0.43	0.31
Employment or own a company	Old age pension	0.52	0.33	0.80	0.44	0.43	0.42	0.40	0.43
Employment or own a company	Long-term sick leave	2.57	1.93	2.16	1.65	1.67	1.95	2.64	2.48
Student or trainee	Long-term sick leave	1.61	1.09	1.52	0.84	0.92	1.33	1.60	1.86
Student or trainee	Old age pension	–	–	0.60	0.18	–	–	–	–
Old age pension	Long-term sick leave	1.46	1.23	0.70	0.61	1.06	1.13	1.76	1.64

*ES*, Effect size, Cohens *d*. An effect is considered to be small if *d* = 0.20–0.49, moderate if *d* = 0.50–0.79, and large if *d* 0.8. ˃

*The effect sizes were calculated by pairwise comparison of the significant differences between the age group of 80+ and all the other age groups, between mandatory education and all the other education subgroups and between the occupation subgroups

### Factor structure

**Fig. 2:**
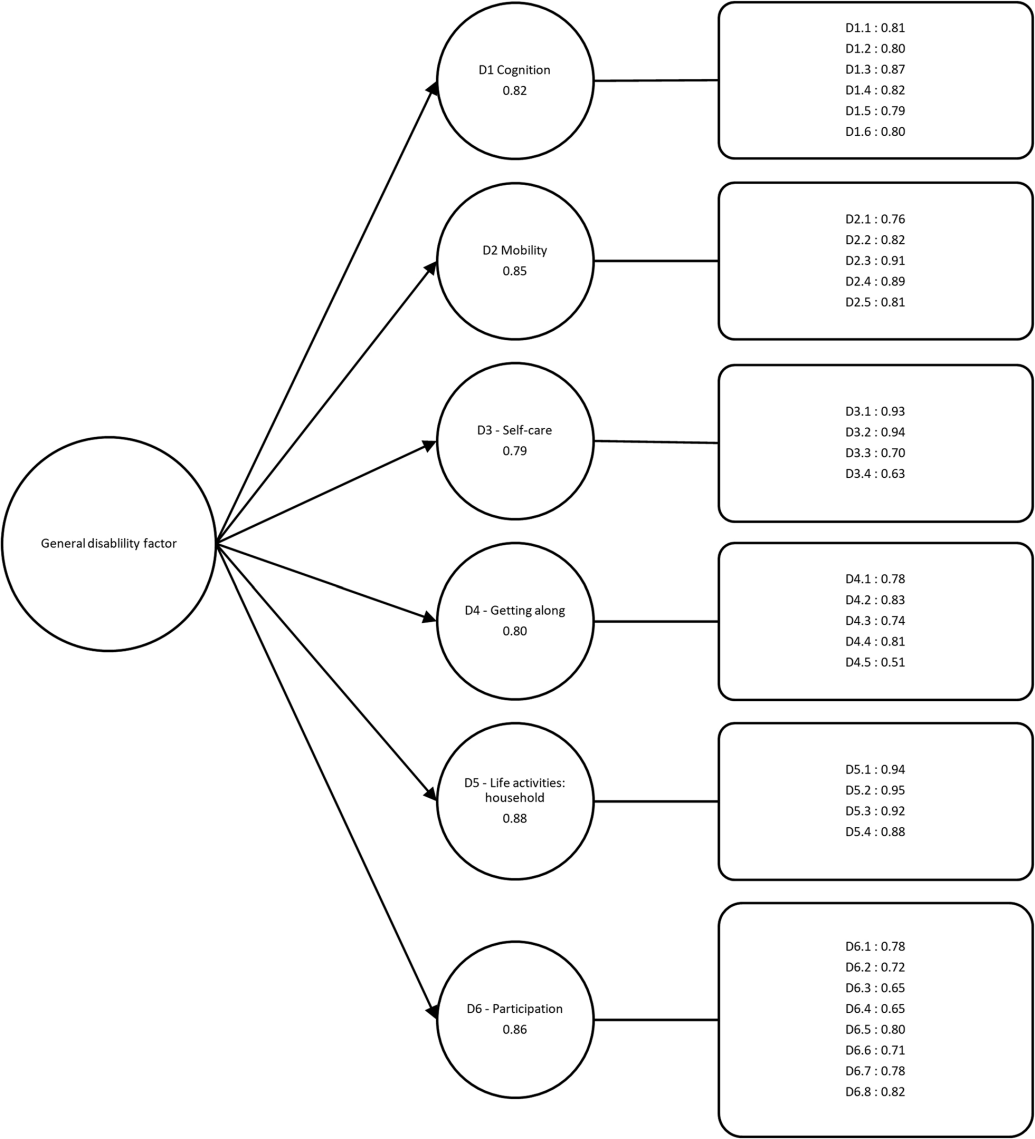
Confirmatory factor analysis. The factor structure of WHODAS 2.0 according to the confirmatory factor analysis with one general disability factor and the six domains as second-order factors. The model fit: Chi-Square (458, N = 3100) = 10,244.5, *p* < 0.0001; RMSEA = 0.08; 90% CI 0.082–0.084; SRMR = 0.06; CFI = 0.89; and TLI = 0.88. The first level consisted of a general disability factor and the second level consisted of the six domains of the instrument. The four items corresponding to the *Life activities: work/study* domain are excluded, hence WHODAS 2.0 with 32-items is used for the confirmatory factor analysis

The CFA results for the proposed second-order factor structure with a general disability factor and the six domains as second-order factors are shown in Fig. 2. The standardized factor coefficients ranged between 0.51 and 0.95, and all the t values were significant (*p* < 0.0001). The RMSEA (0.08; 90% CI 0.082–0.084) and SRMR (0.06) indicated an acceptable fit; however, the CFI (0.89) and TLI (0.88) demonstrated a borderline model fit.

### General population percentiles

In the total sample, the 90th percentile for the WHODAS 2.0 total score was 41.5. In age groups younger than 80 years of age, the 90th percentile were between 34.1 and 42.5, compared to 55.5 in the oldest age group (Additional file 4: Table S4).

## Discussion

To the best of our knowledge this is the first study exploring the psychometric properties of WHODAS 2.0 and the prevalence of disability in a Swedish general population. The Swedish, self-administered, 36-item version of WHODAS 2.0 demonstrated acceptable scale properties with certain weaknesses. Its internal consistency reliability was overall good, its construct validity was acceptable, and the expected disability trends in the known subgroups within the Swedish general population were observed.

The internal consistency reliability of the total score and most of the domains was excellent (alpha ≥ 0.90), except for the *Getting along* and *Self-care* domains*,* which had slightly lower alpha values. Since WHODAS 2.0 is intended for use in clinical practice and for individual assessment, it is important that these alpha values are ≥ 0.90 [16]. The slightly weaker reliability values for *Getting along* and *Self-care* are not unique to our study [8, 9, 12]. The internal consistency reliability of WHODAS 2.0 for somatic rehabilitation patients in Norway showed similar results, as the lowest alpha values in the cited study were found for *Getting along* and *Self-care* [12]. The *Self-care* domain inquires about functioning in basic activities of daily living, activities previously shown to be affected in cases of more severe disability [34]. A literature review discusses whether the lower reliability for the *Self-care* domain can be caused by large floor effects [8]. Floor effects for WHODAS 2.0 have been noted in several studies [8, 12, 35]. The floor effects observed in our study (74.5%) are not surprising, as a large proportion of the general population is expected to have few or no health concerns. However, in clinical populations, the domain score for *Self-care* is probably of substantial interest to rehabilitation clinicians. As such, it is important that this measure be reliable. This question must be considered in a future revision of the WHODAS.

The data partially supported the factor structure of WHODAS 2.0 with one general disability factor and the six domains as second-order factors, which is a common finding in validation studies on the different language versions of WHODAS 2.0 [8, 12, 35–37]. The factor structure of the Swedish version of WHODAS 2.0 in a mixed psychiatric population [17] pointed in the same direction, with borderline fit. These findings indicate a degree of model misfit with partial conceptual overlap between the different domains.

One interesting finding of this study is that the WHODAS 2.0 *Participation* domain is highly correlated with all the RAND-36 subscales. Because of its generally high correlation with the RAND-36, the WHODAS 2.0 *Participation* domain appears to be especially important for the health-related quality of life of adults in the Swedish general population. The correlations that were not aligned with our predefined hypothesis generally showed a higher correlation than had been expected. The moderate to high correlation between the RAND-36 subscales and the WHODAS 2.0 domains can be explained by the fact that all the items within these scales and domains concern health-related difficulties and therefore are not substantially different from one another.

The high proportion of missing answers to items within the Participation domain together with the weak item-scale discriminant validity of the *Getting along* domain item D4.5 (concerning sexual activities) is problematic. Our results indicate that several respondents considered item D4.5 to be not applicable or found it difficult to answer. A large proportion of missing answers corresponding to this item have been reported in several other studies [14, 35, 36, 38] and have been attributed to lack of sexual activity at the time or to the private nature of this item. Item D4.5 was added to the *Getting along* domain based on suggestions from the field interviews and the expert opinion survey conducted during the development of the instrument [9]. This item is thought to contribute with important information despite the high rate of missing data [38]. Others have suggested that the item content should be changed to a more indirect or general question about sexuality or intimate relationships [36]. The Swedish translation was adapted in this way by asking about *being sexually intimate*; however, we still received many missing responses. We consider the rate of missing information as well as the participants’ responses to item D4.5 in the general Swedish population to be valuable information about this specific item. We recommend that future improvements are made and that additional analyses of item D4.5 be conducted in patient populations.

In terms of missing domain scores, the *Getting along* [13, 14, 35] and the *Participation* [14, 35] domains have been observed to have a higher percentage of missing scores compared to the other domains. This phenomenon was also observed in our study, where the *Participation* domain had the highest percentage of missing scores (11.3). The proportion of missing domain scores may partly be affected by the strict rules of WHODAS 2.0 regarding how to address missing data, where only one missing item is allowed in the calculation of the domain score [5]. Regardless, if certain domains have a higher level of missing scores, the items within these domains require special consideration and additional analyses in future studies.

The result that participants ≥ 80 years old had a higher level of disability than the younger participants is in line with an earlier study on the prevalence of disability in Sweden [39]. In this study, a low level of functional impairment was reported in adults younger than 80 years of age, whereas major health changes were observed in adults aged 80–85. Furthermore, ability in self-care (i.e., basic activities of daily life) was observed up to the age of 90, and good performance in life activities (i.e., instrumental activities of daily life) and cognitive status in general was observed until the age of 84 [39]. The same trend with increased level of disability has been observed with the 36-item WHODAS 2.0 in the general population in Spain for individuals aged 80 years [23].

The world norm data presented as general population percentiles included in the WHODAS 2.0 manual [5] are also referred to as a reference for the WHODAS 2.0 score in the DSM-5 [40]. However, these general population norm data were based on an earlier version of the instrument and did not include all 36 items. Our study contributes by adding reference data to the current version of WHODAS 2.0. Furthermore, it adds to the knowledge gap on self-reported disability in the Swedish general population and may function as a more suitable and detailed reference clinically.

The response rate of 43% is a limitation of this study. In addition, the response rate for younger participants was even lower (28%). However, a low response rate is expected, especially among younger adults in the context of population surveys conducted by regular mail. Notably, the response rate for adults older than 60 years was satisfactory (61%). If participation in the study systematically appealed more to certain subgroups of the general population than others, systematic bias in the sample selection may have occurred. For example, females and older adults were represented to a greater extent in our study. However, a relatively large number of participants were included in the study. The stratification of the random sample according to sex and age was comparable to that of the inhabitants of the county in which this study was conducted; the aim was for the sample to reflect the national general population as closely as possible [24]. We therefore consider the composition and the number of participants as sufficient to establish evidence of the validity of the Swedish version of WHODAS 2.0 for the general population.

Another limitation of our study in terms of psychometric evaluation of the Swedish version of WHODAS 2.0 in the general population is the lack of data on the test–retest reliability. Further studies are also needed to evaluate the responsiveness of the Swedish version of WHODAS 2.0 in different populations and settings. Besides the available cutoff score for dysfunction in psychiatric patients in Sweden [19], cut-off scores for other patient populations in Sweden must be investigated to facilitate clinical use of WHODAS 2.0 in Sweden.

## Conclusions

The psychometric properties of the self-administered Swedish 36-item version of the WHODAS 2.0 are comparable to those of other language versions of the instrument. Data of the prevalence of disability in Swedish general population enables normative comparisons of WHODAS 2.0 scores of individuals and groups within clinical practice. However, the instrument has certain limitations that could be improved on in a future revision. The test–retest reliability and responsiveness of the Swedish version of WHODAS 2.0 for different somatic patient populations remain to be evaluated.

## Supplementary Information

**Additional file 1**: **Table S1**. The means and standard deviations (SD) of WHODAS 2.0 domains and total scores by age group

**Additional file 2**: **Table S2**. Means and standard deviations (SD) of WHODAS 2.0 domains and total scores by education level

**Additional file 3**: **Table S3**. Means and standard deviations (SD) of WHODAS 2.0 domains and total scores by main occupation

**Additional file 4**: **Table S4**. General population percentiles of the WHODAS total and domain scores for different age groups

## Data Availability

The dataset used and analysed during the current study are available from the corresponding author on reasonable request.

## Abbreviations

CFA: Confirmatory factor analysis
CFI: Comparative fit index
ES: Effect size
RAND-36: The RAND-36 measure of health-related quality of life
RMSEA: Root mean square error of approximation
SRMR: Standardized root mean square residual
TLI: Tucker Lewis index
WHODAS 2.0: The World Health Organization disability assessment schedule 2.0
