# Supplementary material for: Psychometric evaluation of the WHODAS 2.0 and prevalence of disability in a Swedish general population
Source: J Patient Rep Outcomes. 2023 Apr 5;7:36. doi: 10.1186/s41687-023-00580-0 (PMC10076457; doi:10.1186/s41687-023-00580-0)
Supplement: Supplementary file 1 — Additional file 1: Table S1. The means and standard deviations (SD) of WHODAS 2.0 domains and total scores by age group [file 41687_2023_580_MOESM1_ESM.docx]

**Supplementary Table 1. The means and** **standard deviations (SD) of WHODAS 2.0 domains and total scores by age group**

| WHODAS 2.0 score |  | Total score |  | Cognition |  | Mobility |  | Self-care |  | Getting along |  | Life activities: Household |  | Life activities: Work/study |  | Participation |
| --- | --- | --- | --- | --- | --- | --- | --- | --- | --- | --- | --- | --- | --- | --- | --- | --- |
| Age group | n | Mean (SD) | n | Mean (SD) | n | Mean (SD) | n | Mean (SD) | n | Mean (SD) | n | Mean (SD) | n | Mean  (SD) | n | Mean  (SD) |
| (a) 20-29 | 356 | 14.2 (15.4)^g^ | 381 | 14.8 (17.0)^eg^ | 383 | 7.5 (15.7)^defg^ | 384 | 6.3 (14.7)^g^ | 382 | 17.0 (21.4)^cg^ | 381 | 18.8 (24.6)^g^ | 358 | 15.4 (24.1)^g^ | 363 | 17.2  (18.1)^g^ |
| (b) 30-39 | 456 | 13.0 (16.3)^g^ | 479 | 13.3 (18.1)^g^ | 480 | 7.4 (15.9)^defg^ | 477 | 4.8 (13.0)^g^ | 478 | 15.1 (21.4)^g^ | 481 | 18.0 (25.1)^g^ | 451 | 15.1 (23.3)^g^ | 464 | 16.3  (19.8)^g^ |
| (c) 40-49 | 377 | 11.6 (14.8)^g^ | 399 | 11.3 (17.1)^g^ | 398 | 7.6 (14.9)^defg^ | 400 | 4.3 (12.6)^g^ | 396 | 11.5 (18.3)^afg^ | 398 | 13.7 (22.8)^g^ | 390 | 12.8 (22.4)^g^ | 383 | 15.7  (18.7)^g^ |
| (d) 50-59 | 368 | 14.3 (19.3)^g^ | 388 | 13.9 (20.7)^g^ | 388 | 12.5  (22.3)^abcfg^ | 385 | 6.3 (15.8)^g^ | 388 | 13.1 (22.0)^g^ | 388 | 16.5 (26.1)^g^ | 359 | 13.8 (24.0)^g^ | 374 | 18.3  (22.5)^g^ |
| (e) 60-69 | 535 | 12.8 (16.4)^g^ | 563 | 10.8 (17.6)^ag^ | 565 | 13.5 (21.1)^abcfg^ | 565 | 6.4 (16.2)^g^ | 559 | 13.6 (19.4)^g^ | 568 | 14.3 (23.3)^g^ | 393 | 13.9 (23.5)^g^ | 549 | 16.0  (19.2)^g^ |
| (f) 70-79 | 523 | 14.2 (15.3)^g^ | 570 | 11.7 (16.7)^g^ | 576 | 18.4 (23.6)^h^ | 586 | 7.3 (17.4)^g^ | 573 | 15.6 (18.7)^cg^ | 582 | 16.7 (24.3)^g^ | 256 | 12.7 (18.4)^g^ | 547 | 17.0  (18.2)^g^ |
| (g) 80+ | 374 | 27.9 (20.5)^h^ | 441 | 23.8 (23.5)^h^ | 458 | 35.5 (27.1)^h^ | 458 | 17.6 (26.1)^h^ | 437 | 27.7 (22.8)^h^ | 465 | 35.1 (31.6)^h^ | 157 | 34.8 (28.7)^h^ | 409 | 29.3  (21.9)^h^ |
| Total | 2989 | 15.2 (17.5) | 3221 | 14.0 (19.1) | 3248 | 15.0 (22.7) | 3255 | 7.6 (17.7) | 3213 | 16.2 (21.0) | 3263 | 18.9  (26.3) | 2364 | 15.4  (23.9) | 3089 | 18.4  (20.2) |

One-way ANOVA followed by Tukey’s HSD post hoc test.

^a-g^ = Significant differences (p<0.005, 95% CI) in the pairwise comparisons among the referenced age groups (a, b, c, d, e, f, g)

^h^ = Significant differences (p<0.005, 95% CI) in the pairwise comparisons among all age groups.

Note: a higher score indicates a higher level of disability
