# Supplementary material for: Psychometric evaluation of the WHODAS 2.0 and prevalence of disability in a Swedish general population
Source: J Patient Rep Outcomes. 2023 Apr 5;7:36. doi: 10.1186/s41687-023-00580-0 (PMC10076457; doi:10.1186/s41687-023-00580-0)
Supplement: Supplementary file 2 — Additional file 2: Table S2. Means and standard deviations (SD) of WHODAS 2.0 domains and total scores by education level [file 41687_2023_580_MOESM2_ESM.docx]

**Supplementary Table 2. Means and** **standard deviations (SD) of WHODAS 2.0 domains and total scores by education level**

| WHODAS 2.0 score |  | Total score |  | Cognition |  | Mobility |  | Self-care |  | Getting  along |  | Life activities: Household |  | Life activities: Work/study |  | Participation |
| --- | --- | --- | --- | --- | --- | --- | --- | --- | --- | --- | --- | --- | --- | --- | --- | --- |
| Education | n | Mean (SD) | n | Mean (SD) | n | Mean  (SD) | n | Mean (SD) | n | Mean  (SD) | n | Mean  (SD) | n | Mean (SD) | n | Mean  (SD) |
| Mandatory | 628 | 21.4 (20.7)* | 715 | 19.5 (22.6)* | 732 | 25.6  (27.1)* | 734 | 13.1 (23.6)* | 709 | 22.2  (23.5)* | 736 | 26.3 (30.5)* | 356 | 21.8 (28.1)* | 662 | 23.4  (22.6)* |
| High school | 1008 | 14.1 (16.7)* | 1063 | 14.0 (18.5)* | 1068 | 12.3  (20.1)* | 1068 | 6.2 (15.6) | 1062 | 15.3  (20.7)* | 1071 | 17.1  (25.0) | 903 | 14.5  (23.5) | 1032 | 17.5  (19.7) |
| University | 1013 | 11.8 (14.8)* | 1069 | 10.5 (16.2)* | 1071 | 8.8  (17.6)* | 1073 | 4.5 (12.4) | 1070 | 13.1  (19.3)* | 1073 | 14.7  (22.6) | 895 | 13.1  (21.3) | 1042 | 15.4  (18.0) |
| Total | 2649 | 14.9 (17.5) | 2847 | 14.1 (19.1) | 2871 | 14.4  (22.3) | 2875 | 7.3 (17.3) | 2841 | 16.2  (21.2)* | 2880 | 18.6  (26.1) | 2154 | 15.1  (23.7) | 2736 | 18.1  (20.1) |

One-way ANOVA followed by Tukey’s HSD post hoc test.

*Significant differences (p<0.05, 95% CI) in the pairwise comparisons among all the education subgroups.

Note: a higher score indicates a higher level of disability
