# Supplementary material for: Psychometric evaluation of the WHODAS 2.0 and prevalence of disability in a Swedish general population
Source: J Patient Rep Outcomes. 2023 Apr 5;7:36. doi: 10.1186/s41687-023-00580-0 (PMC10076457; doi:10.1186/s41687-023-00580-0)
Supplement: Supplementary file 3 — Additional file 3: Table S3. Means and standard deviations (SD) of WHODAS 2.0 domains and total scores by main occupation [file 41687_2023_580_MOESM3_ESM.docx]

**Supplementary Table 3. Means and standard deviations (SD) of WHODAS 2.0 domains and total scores by main occupation**

| WHODAS 2.0 score |  | Total score |  | Cognition |  | Mobility |  | Self-care |  | Getting along |  | Life activities: Household |  | Life activities: Work/study |  | Participation |
| --- | --- | --- | --- | --- | --- | --- | --- | --- | --- | --- | --- | --- | --- | --- | --- | --- |
| Occupation | n | Mean (SD) | n | Mean (SD) | n | Mean (SD) | n | Mean (SD) | n | Mean (SD) | n | Mean  (SD) | n | Mean  (SD) | n | Mean  (SD) |
| Employment or own a company | 1463 | 10.3 (13.3)* | 1550 | 10.1 (15.3)* | 1555 | 7.2 (15.0) | 1550 | 3.6 (11.0) | 1547 | 11.3 (17.8)* | 1553 | 12.4 (21.3)* | 1542 | 11.6 (19.9)* | 1491 | 13.3  (16.5)* |
| Student or trainee | 138 | 16.1 (16.9) | 146 | 17.0 (19.2) | 145 | 8.4 (16.5) | 147 | 7.0 (14.8) | 147 | 18.5 (22.8) | 146 | 19.7 (25.0) | 146 | 20.5  (26.0) | 141 | 18.4  (18.4) |
| Old age pension | 1082 | 18.5 (18.6) | 1192 | 15.8 (20.1) | 1220 | 23.6 (26.0)* | 1228 | 10.8 (21.4)* | 1194 | 19.5 (21.0) | 1231 | 22.7 (28.3) | 465 | 20.0  (25.0) | 1137 | 21.2  (20.6) |
| Long-term sick leave | 86 | 45.8 (20.8)* | 95 | 41.2 (26.1)* | 93 | 41.9 (28.7)* | 94 | 24.1 (26.8)* | 90 | 42.5 (30.6)* | 94 | 54.8 (28.5)* | 49 | 65.7 (34.2)* | 92 | 55.0  (21.6)* |
| Total | 2769 | 14.9 (17.4) | 2983 | 13.7 (18.9) | 3013 | 15.0 (22.7) | 3019 | 7.4 (17.3) | 2978 | 15.7 (20.8) | 3024 | 18.5 (26.1) | 2202 | 15.2  (23.5) | 2861 | 18.1  (20.1) |

One-way ANOVA followed by Tukey’s HSD post hoc test.

*Significant differences (p<0.05, 95% CI) in pairwise comparisons among all the other occupation subgroups.

Note: a higher score indicates a higher level of disability.
