# Supplementary material for: Psychometric evaluation of the WHODAS 2.0 and prevalence of disability in a Swedish general population
Source: J Patient Rep Outcomes. 2023 Apr 5;7:36. doi: 10.1186/s41687-023-00580-0 (PMC10076457; doi:10.1186/s41687-023-00580-0)
Supplement: Supplementary file 4 — Additional file 4: Table S4. General population percentiles of the WHODAS total and domain scores for different age groups [file 41687_2023_580_MOESM4_ESM.docx]

**Supplementary Table 4**. General population percentiles of the WHODAS total and domain scores for different age groups

| Age group (years) | Number | Percentiles | | | | | | |
| --- | --- | --- | --- | --- | --- | --- | --- | --- |
|  |  | 5 | 10 | 25 | 50 | 75 | 90 | 95 |
| *WHODAS 2.0 Total score* | | | | | | | | |
| Total sample | 2989 | .0000 | .0000 | 1.8868 | 8.4906 | 22.6415 | 41.5094 | 52.5021 |
| 20-29 | 356 | .0000 | .0000 | 2.8302 | 10.3774 | 19.8113 | 36.7925 | 46.3679 |
| 30-39 | 456 | .0000 | .0000 | .9434 | 6.6038 | 19.8113 | 36.7925 | 47.1698 |
| 40-49 | 377 | .0000 | .0000 | .9434 | 4.7170 | 16.9811 | 34.1263 | 43.5644 |
| 50-59 | 368 | .0000 | .0000 | .9434 | 5.6604 | 20.5189 | 42.5472 | 57.5810 |
| 60-69 | 535 | .0000 | .0000 | 1.8868 | 5.6604 | 16.9811 | 38.6792 | 51.9442 |
| 70-79 | 523 | .0000 | .0000 | 2.1739 | 9.4340 | 19.8113 | 40.2174 | 49.0279 |
| 80 or more | 374 | 1.0870 | 3.2609 | 10.8696 | 25.7793 | 41.3043 | 55.5476 | 64.6893 |
| *Cognition domain score* | | | | | | | | |
| Total sample | 3221 | .0000 | .0000 | .0000 | 5.0000 | 20.0000 | 45.0000 | 55.0000 |
| 20-29 | 381 | .0000 | .0000 | .0000 | 10.0000 | 25.0000 | 40.0000 | 50.0000 |
| 30-39 | 479 | .0000 | .0000 | .0000 | 5.0000 | 20.0000 | 40.0000 | 50.0000 |
| 40-49 | 399 | .0000 | .0000 | .0000 | 5.0000 | 15.0000 | 35.0000 | 55.0000 |
| 50-59 | 388 | .0000 | .0000 | .0000 | 5.0000 | 20.0000 | 50.0000 | 60.0000 |
| 60-69 | 563 | .0000 | .0000 | .0000 | .0000 | 15.0000 | 40.0000 | 50.0000 |
| 70-79 | 570 | .0000 | .0000 | .0000 | 5.0000 | 15.0000 | 40.0000 | 50.0000 |
| 80 or more | 441 | .0000 | .0000 | .0000 | 15.0000 | 40.0000 | 55.0000 | 69.5000 |
| *Mobility domain score* | | | | | | | | |
| Total sample | 3248 | .0000 | .0000 | .0000 | .0000 | 25.0000 | 50.0000 | 68.7500 |
| 20-29 | 383 | .0000 | .0000 | .0000 | .0000 | 6.2500 | 25.0000 | 48.7500 |
| 30-39 | 480 | .0000 | .0000 | .0000 | .0000 | 6.2500 | 25.0000 | 43.7500 |
| 40-49 | 398 | .0000 | .0000 | .0000 | .0000 | 6.2500 | 25.6250 | 43.7500 |
| 50-59 | 388 | .0000 | .0000 | .0000 | .0000 | 12.5000 | 50.0000 | 62.5000 |
| 60-69 | 565 | .0000 | .0000 | .0000 | .0000 | 18.7500 | 50.0000 | 62.5000 |
| 70-79 | 576 | .0000 | .0000 | .0000 | 6.2500 | 31.2500 | 56.2500 | 69.6875 |
| 80 or more | 458 | .0000 | .0000 | 12.5000 | 34.3750 | 56.2500 | 75.0000 | 87.5000 |
| *Self-care domain score* | | | | | | | | |
| Total sample | 3255 | .0000 | .0000 | .0000 | .0000 | 10.0000 | 30.0000 | 50.0000 |
| 20-29 | 384 | .0000 | .0000 | .0000 | .0000 | 10.0000 | 20.0000 | 30.0000 |
| 30-39 | 477 | .0000 | .0000 | .0000 | .0000 | .0000 | 12.0000 | 30.0000 |
| 40-49 | 400 | .0000 | .0000 | .0000 | .0000 | .0000 | 10.0000 | 30.0000 |
| 50-59 | 385 | .0000 | .0000 | .0000 | .0000 | .0000 | 30.0000 | 47.0000 |
| 60-69 | 565 | .0000 | .0000 | .0000 | .0000 | .0000 | 20.0000 | 40.0000 |
| 70-79 | 586 | .0000 | .0000 | .0000 | .0000 | .0000 | 30.0000 | 50.0000 |
| 80 or more | 458 | .0000 | .0000 | .0000 | .0000 | 30.0000 | 60.0000 | 80.0000 |
| *Getting along domain score* | | | | | | | | |
| Total sample | 3213 | .0000 | .0000 | .0000 | 8.3333 | 25.0000 | 50.0000 | 58.3333 |
| 20-29 | 382 | .0000 | .0000 | .0000 | 8.3333 | 33.3333 | 50.0000 | 58.3333 |
| 30-39 | 478 | .0000 | .0000 | .0000 | .0000 | 25.0000 | 42.5000 | 58.7500 |
| 40-49 | 396 | .0000 | .0000 | .0000 | .0000 | 16.6667 | 41.6667 | 50.0000 |
| 50-59 | 388 | .0000 | .0000 | .0000 | .0000 | 16.6667 | 41.6667 | 62.9167 |
| 60-69 | 559 | .0000 | .0000 | .0000 | .0000 | 25.0000 | 41.6667 | 58.3333 |
| 70-79 | 573 | .0000 | .0000 | .0000 | 8.3333 | 25.0000 | 41.6667 | 50.0000 |
| 80 or more | 437 | .0000 | .0000 | 8.3333 | 25.0000 | 41.6667 | 58.3333 | 75.0000 |
| *Life activities: Household domain score* | | | | | | | | |
| Total sample | 3263 | .0000 | .0000 | .0000 | .0000 | 40.0000 | 60.0000 | 80.0000 |
| 20-29 | 381 | .0000 | .0000 | .0000 | 10.0000 | 40.0000 | 50.0000 | 70.0000 |
| 30-39 | 481 | .0000 | .0000 | .0000 | .0000 | 40.0000 | 50.0000 | 70.0000 |
| 40-49 | 398 | .0000 | .0000 | .0000 | .0000 | 20.0000 | 50.0000 | 70.0000 |
| 50-59 | 388 | .0000 | .0000 | .0000 | .0000 | 30.0000 | 60.0000 | 75.5000 |
| 60-69 | 568 | .0000 | .0000 | .0000 | .0000 | 20.0000 | 50.0000 | 70.0000 |
| 70-79 | 582 | .0000 | .0000 | .0000 | .0000 | 30.0000 | 50.0000 | 70.0000 |
| 80 or more | 465 | .0000 | .0000 | .0000 | 30.0000 | 50.0000 | 90.0000 | 100.0000 |
| *Life activities: Work/school domain score* | | | | | | | | |
| Total sample | 2364 | .0000 | .0000 | .0000 | .0000 | 28.5714 | 50.0000 | 71.4286 |
| 20-29 | 358 | .0000 | .0000 | .0000 | .0000 | 21.4286 | 50.0000 | 71.7857 |
| 30-39 | 451 | .0000 | .0000 | .0000 | .0000 | 28.5714 | 50.0000 | 64.2857 |
| 40-49 | 390 | .0000 | .0000 | .0000 | .0000 | 14.2857 | 42.8571 | 71.4286 |
| 50-59 | 359 | .0000 | .0000 | .0000 | .0000 | 21.4286 | 50.0000 | 78.5714 |
| 60-69 | 393 | .0000 | .0000 | .0000 | .0000 | 21.4286 | 50.0000 | 66.4286 |
| 70-79 | 256 | .0000 | .0000 | .0000 | .0000 | 21.4286 | 42.8571 | 50.0000 |
| 80 or more | 157 | .0000 | .0000 | 7.1429 | 35.7143 | 50.0000 | 72.8571 | 100.0000 |
| *Participation domain score* | | | | | | | | |
| Total sample | 3089 | .0000 | .0000 | .0000 | 12.5000 | 29.1667 | 50.0000 | 62.5000 |
| 20-29 | 363 | .0000 | .0000 | .0000 | 12.5000 | 25.0000 | 45.8333 | 54.1667 |
| 30-39 | 464 | .0000 | .0000 | .0000 | 8.3333 | 25.0000 | 45.8333 | 58.3333 |
| 40-49 | 383 | .0000 | .0000 | .0000 | 8.3333 | 25.0000 | 45.8333 | 57.5000 |
| 50-59 | 374 | .0000 | .0000 | .0000 | 8.3333 | 29.1667 | 50.0000 | 70.8333 |
| 60-69 | 549 | .0000 | .0000 | .0000 | 8.3333 | 25.0000 | 45.8333 | 58.3333 |
| 70-79 | 547 | .0000 | .0000 | .0000 | 12.5000 | 25.0000 | 45.8333 | 54.1667 |
| 80 or more | 409 | .0000 | .0000 | 12.5000 | 29.1667 | 45.8333 | 58.3333 | 66.6667 |

IRT-based scoring of the 36-item WHODAS 2.0 for the general Swedish population
